# Efficacy of Quercetin-Sensitized Cisplatin against N-Nitroso-N-Methylurea Induced Testicular Carcinogenesis in Wistar Rats

**DOI:** 10.31557/APJCP.2021.22.1.75

**Published:** 2021-01

**Authors:** Hend HGM El-Diasty, Hassan El-Sayyad, Sherif Refat, Heba A El-Ghaweet

**Affiliations:** 1 *Department of Zoology, Faculty of Science, Mansoura University, Mansoura, Egypt. *; 2 *Department of Medical Oncology, Faculty of Medicine, Mansoura University, Mansoura, Egypt. *

**Keywords:** Testicular cancer, MNU, quercetin, Cisplatin, gene expression

## Abstract

**Background:**

Testicular cancer is a public health problem. The goal of this study was to demonstrate the efficacy of quercetin treatment on N-nitroso-N-methyl-urea (MNU)-induced testicular carcinogenesis alone or in combination with cisplatin-treatment.

**Methods:**

In total 70 adult male albino rats were categorized into six groups, control, quercetin-treatment (10 mg/kg body weight), cisplatin-treatment (2 mg/kg. body weight), cisplatin and quercetin-treatment, MNU-treatment, MNU plus quercetin-treatment and MNU plus quercetin and cisplatin-treatment. Treatment with quercetin and/or cisplatin was performed after 2 months of MNU induced testicular carcinogenesis. The studied groups were euthanized and sacrificed and their testes were examined for gene expression, biochemical, histological and immunohistochemically analysis, inflammation and apoptosis of germ cells.

**Results:**

The fertility of the rats subjected to MNU carcinogenesis was impaired following cisplatin and/or quercetin-treatment. Cisplatin-treatment reduced the fertility rate and improved after quercetin-treatment. Quercetin-treatment decreased the sharp increase in RNA expression of BAX and MPO in both cisplatin-toxicated testes and after MNU carcinogenesis induction. In addition, the testicular levels of testosterone and SOD increased in parallel with depletion of MDA, IL-6, AFP and caspase-3 levels in MNU and/or cisplatin-treatment after –quercetin-treatment. The testicular structure of the cisplatin-treated group recovered their dividing germ and sperm differentiation after-quercetin-treatment. While, there was a great appearance of flourishing germ cell of MNU carcinogenesis post quercetin therapy, there was still a lack of sperm differentiation.

**Conclusion:**

Quercetin-treatment showed increased cisplatin activity and decreased testicular carcinogenesis due to anti-neoplastic and antioxidant activities.

## Introduction

Cancer is a globally significant epidemic. In Egypt, the relative incidence rates 100,000 in 2014 was 166.6 (for both sexes), with the highest incidence being for liver (33.6% Male, 13.5% Female), breast (15.4 % Male, 13.5% Female), and bladder (6.9% (for both sexes) cancer (Ibrahim et al., 2014). Testicular cancer is the most prevalent solid tumor among males 15-34 year-old males, with approximately 8,850 new cases and 410 deaths during 2017 in the United States. Efficient care has increased the survival rate to nearly 97 % of cases. Undescended testis (cryptorchidism) is a common form of testicular cancer. The key initiating factors of this cancer are age, sex and infertility (Baird et al., 2018; Smith et al., 2018). Both genetic and environmental factors contributed to the development of the testicular cancer (Cheng et al., 2018); these factors include the human developmental index (Sadeghi et al., 2016). N-nitosourea-N-methylurea (MNU) is used to cause cancer, such as retinitis pigmentosa (Chen et al., 2016), breast cancer (Soares-Maia et al., 2013; Alvarado et al., 2017; Karia et al., 2018), photoreceptor cell apoptosis (Emoto et al., 2016) and bladder tumors (Yoshida et al., 2018) in animal models. 

The first line of treatment for germ cell testicular cancer is cisplatin-based chemotherapy (Mastrangelo, 2018). The use of surgery and cisplatin- chemotherapy has a >90 % cure rate, while some patients suffer late recurrence (Cheng et al., 2018).

Quercetin-treatment in combination with cisplatin modulated gene expression of cyclins and cyclin dependent kinases is also used. In addition, the upregulation of genes involved in JNK, p38 and MAPK/ERK pathways coincides with decreased ERK phosphorylations (Demiroglu-Zergeroglu et al., 2016). Flavonoids are polyphenolic compounds which are effective therapeutic agents against different types of cancer (Brito et al., 2015). 

Curcumin, quercetin, and allicin are used to treat gastric cancer (Haghi et al., 2017). Taxifolin is a flavonoid with a chemo-preventive agent against prostate cancer which works by suppressing androgen secretion in Leydig cells (Ge et al., 2018). Quercetin, which is a natural defensive bioflavonoid, has antioxidant, anti-inflammatory, anti-proliferative, and anti-angiogenic properties and is effective in cancer - treatment (Men et al., 2014). 

However, a primary cause of cisplatin resistance in malignancies is the expression of Wnt16 in cisplatin-damaged tumor-associated fibroblasts. This has led authors to use natural anti-fibrotic compounds to downregulate Wnt16 expression, improving the antitumor effect of cisplatin nanoparticles (Hu et al., 2017). 

The present study aimed to illustrate the therapeutic potential of quercetin and/ or cisplatin in the treatment MNU induced testicular carcinogenesis.

## Materials and Methods

*Animal studies and ethical guidelines*

This study was conducted according to the guidelines of the National Institute of Health for the use of laboratory animals (NIH Publication No, 8523, revised 1996) and confirmed by the local Experimental Animal Ethical Committee of Mansoura University, Egypt. A total of seventy adult male albino rats weighing approximately 100±10 g body weight were obtained from Hellwan Breeding Farm, Ministry of Health, Egypt and used for experimentation. They were allowed to acclimatize for 15 days before experimentation. Free access to food and water was allowed and they were kept well-ventilated with 12 hr light and dark cycle.

*Induction of testicular carcinogenesis*

This was carried using methyl nitroso-urea (MNU) (Sigma-Aldrish Company). MNU is the traditional precursor in the synthesis of diazomethane. The applied dose 30mg/kg body weight is dissolved in saline solution (Chen et al., 2016). Three doses were administered intraperitoneally, once per week for consecutive three weeks and follow up for 3 months.

*Cisplatin-treatment*

Cisplatin is an antineoplastic medication used to treat a variety of cancers including testicular cancer. It has a formula of Pt (NH3)_2_Cl_2_ and was developed by the EMIC pharmaceutical industry (Cairo Egypt). It was diluted with ringer solution and two doses of 2mg/kg body weight were intraperitoneally injected once a week after the second month of MNU inducing carcinogenesis. 

*Quercetin-treatment*

Quercetin is a plant flavonoid with chemical formula C15H10O7. It was obtained from the international laboratory USA and orally administered every other day at doses of 10 mg/kg body weight dissolved in saline solution for two months starting with the second month-treatment of MNU-induction.

*Experimental animals*

**Figure 1:**
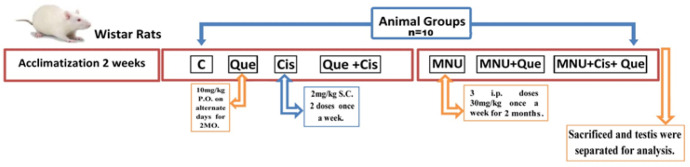
Chart Illustrating Experimental Design

Seventy Wister albino rats (Rattus norvegicus) weighing approximately 100 g, were divided into seven groups (n = 10) such as control, quercetin-treatment (10 mg/kg body weight), cisplatin-treatment (2 mg/kg.body weight), cisplatin and quercetin-treatment, MNU-treatment, MNU plus quercetin-treatment and MNU plus quercetin and cisplatin-treatment. After two months induction of testicular carcinogenesis using MNU, treatment was conducted out with quercetin and or cisplatin. In addition, the fertility of the male rats of the studied groups was achieved by mating each male with fertile female and determining the fertility rate and number of newborns. After two extra-months- treatment (four months), the studied groups were euthanized using chloral hydrate (300mg/kg body weight) and sacrificed. The testes were dissected and processed for the following investigations; detailed below (Figure 1). 

*Body and testicular weight*

Changes in percentage of body weight were determined after two months of induction carcinogenesis. Simultaneously, the absolute and relative testes weights were recorded at four months after treatment. 

*Histological investigation*

The specimens were fixed in 10 % phosphate buffered formalin (pH 7.4), dehydrated in higher grades of ethyl alcohol, cleared in xylene and mounted in melted paraplast at 58-62 ºC. Histological sections of 5 µm were cut, stained with hematoxylin and eosin and examined under a bright field light microscope. 

*Immunohistochemistry for Caspase-3, TNF-α, PCNA and P53*

Samples of 5 μm of formalin-fixed, paraffin-embedded testis were mounted on polylysine-coated glass slides. This was followed by developing, dewaxed tissue sections in xylene and rehydrating in a descending series of alcohol. The tissue sections were incubated in 3% H_2_O_2_ for 10 min to reduce endogenous peroxidase activity. The tissue sections were placed in digested media composed of 0.05 % trypsin (pH 7.8) for 15 min at 37°C and incubated with the primary monoclonal mouse antibody of caspase-3 (DAKO, clone MIB5, 1:50, mouse) and primary antibody against (TNF-α) antibody, proliferating cell nuclear antigen (PCNA) and (P53) antibody at 1:50 overnight at 4°C. After washing, the slides were incubated with a secondary biotin linked anti-mouse antibody for 50 min at room temperature; and with the streptavidin-peroxidase complex for 50 min. Sections were then washed and incubated with developing solution (diaminobenzidine-hydrogen peroxide; DAKO), and counterstained with hematoxylin. The immune reaction was visualized as brown nuclear or cytoplasmic labeling counterstained with hematoxylin. Sections incubated with 1% nonimmune serum phosphate buffer solution (PBS) solution served as negative controls. Finally, the histological sections were examined under a bright field light Olympus microscope with a digital canon camera. In addition, slides captured for image analysis were photographed using an Olympus^®^ digital camera mounted on Olympus^®^ microscope with 1/2 X photo adaptor, at 40 X objective. The resulting images were analyzed on an Intel^®^ Core 5^®^ based computer using Video Test morphology^®^ software (Russia) and the percentage area was measured and recorded.

*Biochemical assays*

The testes of the studied groups were weighed, homogenized with phosphate buffer and centrifuged. The supernatants were separated and kept in refrigerator. 

*Determination of IL6, AFP, caspase-3 and testosterone*

Interlukin-6 was assayed at 450 nm using and enzyme-linked immunosorbent assay (ELISA) kit from Bio Vision catalog No.K4145-100. Alpha-fetoprotein (AFP) was assayed at 450 nm using an ELISA Kit from Bio Vision catalog No. SEA153Ra-96). Testosterone was assayed at 450 nm using ELISA Kit of Bio Vision catalog No. (#K7418-100). Caspase-3 was determined colorimetrically using a Stressgen kit (catalog No. 907-013). The method is based on the addition of the caspase 3 specific peptide to the lysed testicular tissues and the color of the reaction is developed through the addition of p-nitroaniline (pNA). Quantitative colored spectrophotometrically was measured at a wavelength of 405 nm.

*Determination of SOD and MDA*

Superoxide dismutase (SOD) was determined following Niskikimi et al., (1972) and based on the inhibition of nitro blue tetrazolium (NBT). The reduction of NBT by superoxide radicals to blue colored formazan was assayed at 560 nm. Malondialdhyde (MDA) was determined following Ohkawa et al., (1982). The testicular MDA level was determined based on protein concentrations of samples, estimated at 532 nm and expressed as η mol/ mg protein.

*Gene expression*

Testicular mRNA was extracted according to the reagent protocol (Thermo scientific Gene JET RNA Co, America). RNA was assessed using electrophoresis on 2% agarose gel stained with ethidium bromide and the optical density of the RNA was determined. In addition, the RNA concentration was determined using the spectrophotometry method. A reverse transcription kit (Qiagen) was used to remove of DNA contamination and for the subsequent cDNA synthesis. Complementary DNA (cDNA) was synthesized using RevertAid First Strand cDNA Synthesis Kit (Thermo Co, BIO-6505, USA) following the manufacturer’s protocol. The temperatures and the timings used for each cycle depend on a wide variety of parameters, such as: the enzyme used to synthesize the DNA, the concentration of divalent ions and deoxy ribonucleotides (dNTPs) in the reaction and the bonding temperature of the primers (Abdraboh et al., 2018).

*Statistical analysis*

Data were expressed as a mean ± standard error (SE). Statistical analyses were conducted using SPSS (version 13) software package for Windows with one-way post-hoc analysis of variance (ANOVA), comparing the variance between the analyzed groups and P<0.05 was considered statistically significant.

## Results

*Body weight gain and absolute and relative testis weight*

**Figure 2:**
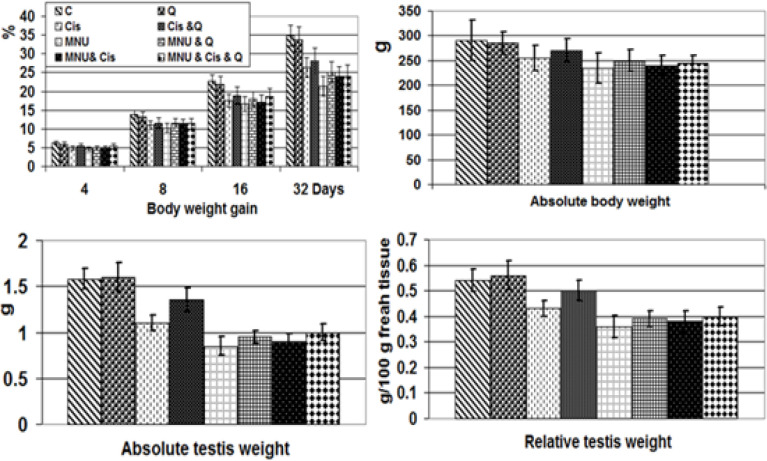
Percentages of Body Weight Gain and Absolute Body Weight, Testis Weight and Relative Testis Weight of Male Rats Subjected to MNU Carcinogenesis and Treated with Cisplatin and/or Quercetin. Each result represents the mean + SD (n=7),* Significant at P<0.05. Abbreviations; C, control; Cis, cisplatin; Cis +Q, cisplatin and quercetin; MNU, methyl nitroso urea; MNU+Q; methyl nitroso urea and quercetin; MNU+Q+Cis; methyl nitroso urea plus quercetin plus cisplatin

Compared to the control, the gain in body weight in the MNU carcinogenesis group was significantly less than in the cisplatin-treated group. Quercetin-treatment had less of an effect on MNU carcinogenesis than the cisplatin- treated group. The MNU carcinogenesis group demonstrated a decreased absolute and relative testis weight compared with the cisplatin-toxicated group and varied markedly from the control values (Figure 2). 

*Morphometric assessment of testicular structure*

The cisplatin intoxicated group showed a significant increase in the total number of inactive seminiferous tubules (which are those lacking sperms) compared with the control group. However, the co-administration of quercetin to the cisplatin-treated group increased the overall number of active seminiferous tubules but there were still less than for the control. In the MNU-treatment, there was a significant decrease in the diameter of the seminiferous tubules associated with almost missing germ cells compared with the control at P < 0.05. However, quercetin-treatment MNU carcinogenesis led to the regeneration of a small group of spermatogonial cell populations coinciding with a less improved seminiferous tubular diameter. Co-administration of cisplatin and quercetin to MNU carcinogenesis possessed a moderate improvement but was less variable than the quercetin and MNU experimental groups. The mean diameter of the seminiferous tubules was markedly increased in the MNU-intoxicated group but it was still significantly decreased compared with the control. MNU and /or cisplatin, showed a significant reduction in germ cell height and a moderate improved after quercetin treatment, but the germ cells were still less smaller than those in the control group (Table 1).

*Histopathological observations*

**Figure 3:**
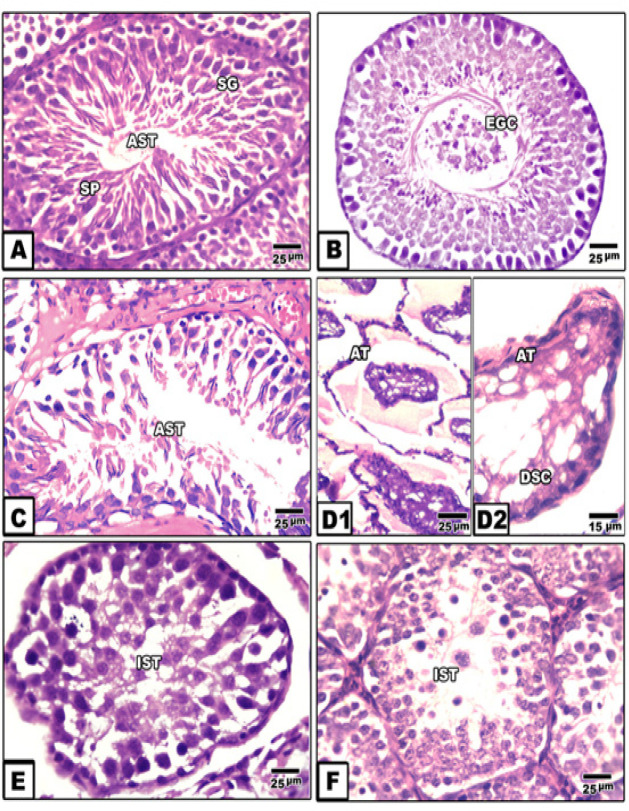
Photomicrographs of Histological Cross Section of Rat Testis. A. Control showing active ST. B. Cisplatin intoxication showing reduction of germ cells and exfoliation of some within tubular lumina. C. Quercetin therapy of cisplatin intoxication showing regenerated spermatogenic cells. D&D1. MNU carcinogenesis showing atrophy and degeneration of germ cells. E. MNU carcinogenesis treated with quercetin showing regenerated spematogonial and spermatocyte cells. F. MNU carcinogenesis treated with cisplatin and quercetin showing regenerated germ cells but no presence of sperm. Abbreviations; ACT, active seminiferous tubules; AT, atrophied tubule; DSC, degenerated spermatocyte; EGC, exfoliation of germ cells; IST,imactive seminiferous tubule; SG, spermatogonia; Sg, spermatocyte

The cisplatin treated group exhibited massive degeneration of the spermatogenic cells. Some seminiferous tubules appeared sclerotic. In addition vacuolation of spermatogonial cells and exfoliation of germ cells within the tubular lumina were observed. There was a massive reduction in spermatogenic cells and missing sperms compared with the control group (Figure 3AB). 

Co-administration of quercetin to the cisplatin-toxicated group resulted in the development of newly regenerated spermatogenic cell populations. However a small edematous lesions and congested blood vessels as well as only multinucleated giant cells were observed. There was a lack of sperm compared with in the control (Figure 3 C). 

Compared with the control and cisplatin-treated groups, the MNU carcinogenesis group showed a marked increase of atrophic seminiferous tubules with folded basal lamina. Few scattered germ cells were detected. A dense aggregation of inflammatory cells, fibroblasts and suspected tumor cells were observed among the intertubular connective tissues (Figure 3 D1 and D2). Quercetin-treatment to the MNU carcinogenesis group showed regeneration of small numbers of spermatogonial and spermatocyte cells. The intertubular fibrous tissues were substantially reduced and suspected tumor cell numbers decreased but hyalinization was still detected. Inflammatory and mutagenic cell number also decreases (Figure 3 E). Quercetin and cisplatin-treatment of the MNU-carcinogenesis group showed some degrees of improvement but seminiferous tubules still appeared in the inactive form and lacked spermatid and sperm differentiation. There was hyalinization of the intertubular tissues (Figure 3 F).

*Immunohistochemistry of testes*

**Figure 4:**
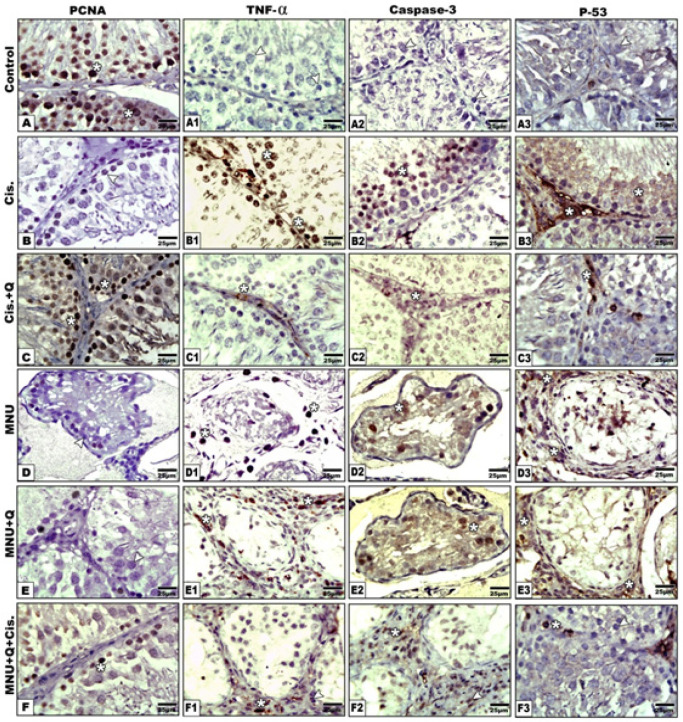
Photomicrographs of Formalin Fixed Testis Immunohistochemical Stain with PCNA (A-F), TNF-α (a1-F1), Caspase 3 (A2-F2) and PF3(A3-F3) Vertical. Note increased immunostaining of PCNA in control and improvement post quercetin-treatment of cisplatin and MNU carcinogenesis. TNF-α showing overexpression in cisplatin intoxication and MNU carcinogenesis and improved in quercetin-treated groups. Caspase intoxication showing increased immune reaction in cisplatin intoxication and MNU carcinogenesis and improved in quercetin-treated studied groups. P53 showing increased immune staining in cisplatin intoxication and MNU carcinogenesis and improved in quercetin-treated studied groups

**Figure 5:**
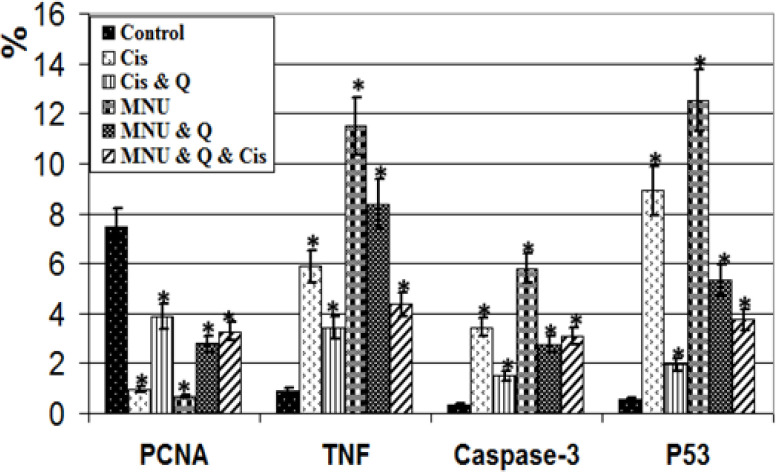
Image Analysis of Male Testis Subjected to MNU Carcinogenesis and Treated Treated with Cisplatin and/or Quercetin. PCNA showing significant decreased image reaction in cisplatin and MNU carcinogens and/or quercetin-treatment. TNF-α expressing inflammation showing significant increase of image analysis in cisplatin and MNU carcinogens and/or quercetin-treatment. Caspase-3 expressing apoptosis showing significant increase image analysis cisplatin and MNU carcinogens and/or quercetin-treatment. P53 reflecting mutagenicity showing significant increase image analysis cisplatin and MNU carcinogens and/or quercetin-treatment. Stare mean significant at P ‹ 0.05

Compared with the increased expression of proliferating cell nuclear antigen (PCNA) in the control and quercetin-treated group, treatment with either cisplatin or MNU carcinogenesis showed a significant decrease of the immunohistochemical reaction. However, quercetin-treatment with either MNU-carcinogenesis group and/or cisplatin-treatment showed a moderate increase of PCNA immunostaining (Figure 4). Image analysis of the PCNA showed a marked reduction of the proportion of immunostaining activity in both the cisplatin toxicated group and MNU carcinogenesis (Figure 5).

In both testes intoxicated with cisplatin and MNU carcinogenesis, overexpression of caspase-3 reflecting cell death was observed in the spermatogenic cells. Co-administration of quercetin to the cisplatin-toxicated group decreased the immunostaining of caspase-3. In addition, caspase-3 immunoreactivity was increased in the sparse distributed germ cells of MNU carcinogenesis and in those treated with cisplatin-treatment (Figure 4). Image analysis showed a significant increase of caspase 3 in the cisplatin and MNU carcinogenesis groups compared with the other studied groups (Figure 5). 

With respect to TNF-α immunohistochemistry, both the cisplatin intoxicated group and MNU carcinogenesis group demonstrated increased immunostaining reflecting increased inflammation. However, co-administration of quercetin to cisplatin-treatment showed a decreased TNF-α immunohistochemical reaction. However, quercetin-treatment decreased the immunoreaction of TNF-α in the MNU carcinogenesis and /or cisplatin-treatment (Figure 4). Following image analysis, TNF-α showed a significant increase in the immune reaction in the cisplatin intoxicated testis and MNU carcinogenesis compared with the other studied groups (Figure 5). 

Compared to the control, the testis overexpressed P53 in both the cisplatin-treated group and the MNU carcinogenesis group. However, co-administration of quercetin to the cisplatin-treated group decreased the expression of P53. In addition, decreased immunostaining was observed in the MNU carcinogenesis and/or cisplatin intoxication and moderately improved in the after quercetin-treatment (Figure 4). Image analysis of P53 revealed a significant increase in the cisplatin intoxicated testis and MNU carcinogenesis compared with the other studied groups (Figure 5). 

*Biochemical investigations*

In the experimental cisplatin intoxicated group and MNU carcinogenesis group, there was a significant decrease of testicular testosterone and SOD coinciding with an increase in the content of MDA, IL-6, AFP and caspase-3 compared with control group. Also, co-administration of quercetin to either the cisplatin-intoxicated group or MNU carcinogenesis group up-regulated testosterone and SOD and consequently decreased the levels of MDA, IL-6, AFP and caspase-3 (Table 2). 

*Gene expression of BAX, BCL2, MPOandSOD by real time PCR*

**Figure 6:**
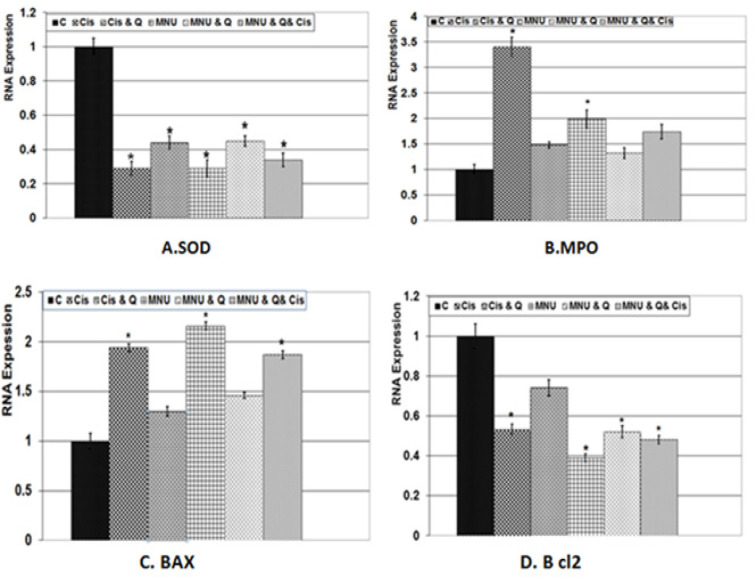
RNA Expression of Testis of Male Rat Subjected to MNU Carcinogenesis Testicular Tumor and Treated with Cisplatin and/or Quercetin Treatment. A. SOD gene expression. B. MPO gene expressio. C. BAX gene expression. D. Bcl2 gene expression. Each result represents the mean ± SD (n=4). Stare mean significant at P ‹ 0.05

From figures (6 A-D), quantitative RNA expressions of BAX, bcl2, MPO, SOD were assayed in testes of rats subjected to MNU carcinogenesis and treated with either cisplatin or quercetin. Gene expression of BAX and MPO were markedly increased in both cisplatin toxicated and MNU carcinogenesis groups. Their levels were markedly decreased in the studied group treated with quercetin and/or cisplatin treatment. Quercetin phytotherapy significantly improved gene expression compared with the cisplatin-treatment. However, the gene expression of both SOD and bcl2 were highly increased in both the control and quercetin-treated groups. The gene expression of SOD and bcl2 were significantly increased in both cisplatin of intoxicated group and MNU carcinogenesis group. Co-administration of quercetin and/or cisplatin to MNU carcinogenesis increased gene expression of SOD and bcl2, but their levels were still below normal values.

**Table 1:** Morphometric Assessment of Testicular Structure Subjected to MNU Carcinogenesis and Treated with Cisplatin and/or Quercetin

	C	Cis	Cis + Q	MNU	MNU + Q	MNU + Q + Cis
Total Numbers	22 (100%)	31 (140%)	25 (114%)	30 (136%)	28 (127%)	27 (123%)
Active ST		63.90%	74.40%	0		
Inactive ST	0	36.10%	25.60%			
Mean diameter of ST (µ)	292 + 0.23	240 + 0.20	272 + 0.05	221 + 0.22	238 + 0.17	242 + 0.09
Germ cell height (µ)	96 + 0.09	75 + 0.08	80 + 0.05	22 + 0.11	38 + 0.01	34 + 0.01

Each result represents the mean + SD (n=7),* Significant at P<0.05. Abbreviations; C, control; Cis, cisplatin; Cis +Q, cisplatin and quercetin; MNU, methyl nitroso urea; MNU+Q; methyl nitroso urea and quercetin; MNU+Q+Cis; methyl nitroso urea plus quercetin plus cisplatin.

**Table 2:** Biochemical Markers of Testicular Tumor Treated with Cisplatin and/or Quercetin Treatment

	T (ng/mg protein)	SOD (U/mg Protein)	MDA (nmol/mg protein)	IL6 (Pg/mg protein)	AFP (ng/mg protein)	Casp-3 (ng/mg)
C	3.35 + 0.5	17.05 + 0.9	1.19 + 0.2	16.25 + 1.8	1.05 + 0.2	3.15 + 0.7
Cis	2.58 + 0.5*	11.15 + 0.9*	1.91 + 0.2*	23.89 + 2.8*	2.59 + 0.3*	5.44 +0.8*
Cis+Q	2.69 + 0.5*	12.51 + 0.8*	1.52 + 0.3*	22.13 + 3.8*	2.31 + 0.3*	5.01 +0.5*
MNU	2.32 + 0.5	14.12 + 0.8	1.83 + 0.2	25.32 + 2.4	3.31 + 0.3*	4.0 + 0.4
MNU+Q	3.0 + 0.4	14.5 + 0.8	1.54 + 0.2	18.16 + 1.8*	2.24 + 0.2*	3.82 + 0.5
MNU+Q+Cis	2.89 + 0.4	12.01 + 0.9*	1.31 + 0.3*	20.55 + 2.8*	2.53 + 0.3*	4.52 +0.4*
F-Test	2.82	57.9	232.18	85.28	28.47	15.06
P<0.05	S.	S.	S.	S.	S.	S.

Each result represents the mean + SD (n=7). Stars means significant at P<0.05. Abbreviations; C, control; Caspase-3, cysteine-aspartic acid proteas 3; Q, quercetin; SOD, superoxide dismutase enzyme; Cis, cisplatin; MDA, malondialdehyde; Cis+Q, cisplatin and quercetin; IL6, interlukin 6; MNU, methyl nitroso urea; T, testosterone; MNU+Q, methyl nitroso urea and quercetin; AFP, alpha fetoprotein; MNU+Q+Cis, methyl nitroso urea and quercetin and cisplatin.

## Discussion

In the present work, cisplatin-treatment and/or MNU carcinogenesis significantly decreased gains in body weight and both absolute and relative testis weigh. The testicular tissues are highly susceptible to cisplatin-treatment; resulting in seminiferous tubule atrophy and loss of germ cell contents. This has been confirmed by increased immunohistochemical reaction of caspase-3 parallel to decreased cell proliferation (PCNA) and increased cell death (caspase 3). The observed findings confirmed the work of Adejuwon et al., (2015) and Prihatno et al., (2018) following the analysis of testicular dysfunction of rat’s after-cisplatin-treatment.

Increased testicular cell death attenuated the increased immunohistochemical staining of TNF-α reflecting inflammation which triggered an increase of P53 immunohistochemistry (which is a tumor suppressor gene) that reflects cell death through an increase in caspase 3 immunohistochemistry as well as increased gene expression of BAX and MPO and a decrease in Bcl2. This activated testicular cell damage announced by increased testicular levels of caspase-3 biochemically through immunohistochemically, manifesting in cell death (El-Sisi et al., 2017).

The present findings are consistent with those reported by Chipuk et al., (2004) who reported that p53 promoted BAX associated cell death.

There was a detected increase in immune reaction of TNF-α advancing the increase in testicular damage leading to a decrease in antioxidant defense in both SOD activity and gene expression. At the same time, the testicular MDA contents of cisplatin-treated rats increased reflecting the increase in oxidative stress. The present findings are consistent with those reported by Yucel et al., (2019) following assessments of all-trans retinoic acid in cisplatin-induced testicular damage.

The observed drastic testicular damage was accompanied by decreased SOD activity and increase MDA content reflecting an increase in oxidative stress. These manifested in a decreased oxidative defense against liberated free radicals which increased the MDA level (Schaalan et al., 2018; Adelakun et al., 2018; Liu et al., 2019). Observed testicular cell damage may result from the production of free oxygen species and decreases the SOD antioxidant, leading to the reduction of the oxidant/antioxidant balance which intern depleted the testosterone level involved in the proliferation of the germ cells.

 Cisplatin intoxication has also been shown to decrease the testosterone blood levels (Tian et al., 2018) and both testosterone level and activity of testicular 3β-hydroxysteroid dehydrogenase and 17β-hydroxysteroid dehydrogenase (Reddy et al., 2016), resulting in testicular impairment and reducing fertility of male rats thereby decreasing the number of newborn. 

Quercetin therapy of cisplatin intoxicated rats improved the testicular structure assessed by comparative increase of tubular diameter, increase of both cell density and germ cell height. This was verified by decreasing the testicular level of both caspase-3 and MDA and increasing the production of SOD and the testicular testosterone level suggesting a decrease of oxidative stress and damage to the testicular cells.

Such results have been confirmed by natural products such as resveratrol (Reddy et al., 2016) and grape seed procyanidins extract (Tian et al., 2018) have been found to boost testicular damage and the depletion of testosterone associated with cisplatin toxicities through the increase in mRNA and protein levels of testosterone synthetase in rat testes. Quercetin metabolizes to quercetin-3-O-glucuronide (Baral et al., 2017) and it displays lipophilic activity that allows molecules to penetrate through cell membranes and have detoxification and antioxidant activities through scavenging superoxide free radicals in the human body (Zhang et al., 2006) through electron-transfer processes (Vásquez-Espinal et al., 2019; Abarikwu et al., 2012) .

However, the MNU associated testicular cancer increased the number of intertubular pleomorphic carcinogenic cells which was correlated with inflammatory cell infiltration along with increased collagen deposition and hyalinization of intertubular tissue. Such results were confirmed by over expression of the proinflammatory markers IL6 and biochemically TNF- α immunohistochemically.

The presence of polymorph nuclear cells within the intertubular tissue was reflected by an increase in testicular α-fetoprotein (AFP) which is a 70 kilo Dalton single-chain glycoprotein predicts the development of testicular cancer (Iwatsuki et al., 2016) .

In addition, MNU carcinogenesis caused pronounced loss of the germ cells within the seminiferous tubules and this was confirmed by an increase in caspase-3 and MDA which was parallel to the reduction of antioxidant SOD activity.

The present findings are consistent with Shamila et al., (2014) who reported the progression of cancerous cell which included the reduction of apoptotic protein and a decrease in antioxidant enzymes. It was also found that testicular cancer is linked with Leydig cell dysfunction (Bandak et al., 2018) which is involved in testosterone secretion.

The mechanism by which alkylating agents (MNU) produce tumors in experimental animals was determined via a DNA alkylation product, O6-alkylguanine, which initiates malignant transformation in several experimental carcinogenesis systems (Magison et al., 2002). 

We found that, cisplatin therapy of MNU carcinogenesis resulted in the reduction of collagenous condensation of intertubular connective tissue and a decrease in cancerous and inflammatory cells. 

The present work agrees with the work of Richie (2005) who reported high sensitivity to cisplatin-treatment of the in vitro culture of neoplastic germ cell lines through the mediation of p53-independent apoptosis-inducing effects on malignant human testicular germ cells. Patients with testicular cancer should be cured with the co- treatment of cisplatin + vinblastine + bleomycin (Einhorn, 2003) or cisplatin alone (Oing et al., 2018).

However, an increased optimum of testicular improvement was observed in quercetin- treatment of MNU carcinogenesis and/or cisplatin treatment. 

The observed increase in the inflammation of MNU testicular carcinogenesis was assessed by increased TNF-α, p53 and caspase 3 immunohistochemistry as well as Il6 and caspase 3 biochemically. The degree of inflammation was alleviated by quercetin- treatment of the MNU.

These anti-inflammatory functions of quercetin resulted from either inhibiting the release of cytokines, decreasing the production of COX and LOX (Dower et al., 2015; Perez-Cano and Castell, 2016) or binding with the active site of lipoxygenase leading to degradation (Borbulevych et al., 2017). Also, quercetin is metabolized into quercetin-3-O-glucuronide associated with increased cleavage of caspase-3, PARP and reduced proliferation in vivo and in vitro studies of neuronal cells (Baral et al., 2017). The decreased in both carcinogenesis and the testicular level of α fetoprotein were attributed to its capacity to induce apoptosis in cancer cell lines such as CT-26, LNCaP, MOLT-4 and Raji (Hashemzaei et al., 2017) and consequently decreased cell proliferation, inhibiting angiogenesis and metastasis progression (Tang et al., 2020). Also, its antitumor activity resulted from increase the expression of pro-apoptotic proteins which was recorded in HL-60 acute myeloid leukemia cell, A375SM melanoma cell and A2780S ovarian cancer cell apoptosis (Kim et al., 2019).

In conclusion, quercetin showed therapeutic potential against testicular cancer and its combination with the cisplatin-treatment resolved its cytotoxicity and improved the antioxidant activity of the testicular cells.

## Authors contribution

Hend HGM El-Diasty, done the practical work under the supervision of the authors and the aid of Dr. Heba A. El-Ghawet. Hassan El-Sayyad editing tables and figures and construct the manuscript in final form. Sherif Refat, done the statistical analysis and investigates the clinical symptoms and histological investigations. Heba A El-Ghaweet, categorized the data, investigating the histopathology and immunohistochemistry and revised the data.

## Conflict of interest statement

The authors confirm that there are no conflicts of interest associated with this publication and there has been no significant financial support for this work that could have influenced its outcome. 
